# Band Engineering Induced by Sulphur Vacancies in MoS_2_/g-C_3_N_4_ or Selective CO_2_ Photoreduction to CH_3_OH

**DOI:** 10.3390/nano15171294

**Published:** 2025-08-22

**Authors:** Shicheng Liu, Junbo Yu, Xiangyu Chen, Na Li, Qulan Zhou

**Affiliations:** Multiphase Flow in Power Engineering, School of Energy and Power Engineering, Xi’an Jiaotong University, Xi’an 710049, China; scliu98@stu.xjtu.edu.cn (S.L.); yyjb6666@stu.xjtu.edu.cn (J.Y.); chenxiangyu8124@stu.xjtu.edu.cn (X.C.); lyna@mail.xjtu.edu.cn (N.L.)

**Keywords:** photocatalytic CO_2_ reduction, S-scheme heterojunction, sulphur vacancies, methanol selectivity, band alignment modulation

## Abstract

Developing photocatalysts with both high efficiency and reaction pathway selectivity is essential for achieving efficient and sustainable CO_2_ conversion. By incorporating sulphur vacancies into MoS_2_, an S-scheme heterojunction photocatalyst (MoS_2_-SVs/g-C_3_N_4_) was developed, achieving efficient and selective CO_2_ photoreduction to CH_3_OH. The structural and photoelectronic characterisation of the system shows that the heterogeneous interface between MoS_2_ and g-C_3_N_4_ is in close contact. The introduction of SVs effectively modulates the electronic structure and surface activity of MoS_2_, which in turn enhances the CO_2_ reduction performance. Optical and electronic structure analyses reveal that the heterojunction promotes favourable band alignment and interfacial electric potential gradients, which together suppress charge recombination and enhance directional carrier separation. Under irradiation, the MoS_2_-SVs/g-C_3_N_4_ photocatalyst exhibited outstanding photocatalytic CH_3_OH production with a yield of 10.06 μmol·h^−1^·g^−1^, significantly surpassing the performance of control samples while demonstrating excellent product selectivity and remarkable stability. Mechanistic studies further verify that vacancy-induced energy band modulation with Fermi energy level enhancement significantly reduces the multi-electron transfer barrier, thus preferentially driving the CH_3_OH generation pathway. This work proposes a universal structural design strategy that synergistically coordinates vacancy engineering with band structure modulation, establishing both theoretical principles and practical methodologies for developing selective multi-electron CO_2_ reduction systems.

## 1. Introduction

The solar-powered photocatalytic CO_2_ reduction reaction provides a promising technology for converting greenhouse gases into high-value-added hydrocarbons (CH_3_OH), which not only contributes to climate change mitigation but also provides a pathway for renewable energy storage [1,2,3]. However, poor product selectivity is a major problem in photocatalytic CO_2_ reduction reactions. The availability of multiple protons and electrons leads to uncontrolled selectivity of the products generated by the reaction (CO, HCOOH and CH_3_OH) [4,5,6]. Kinetic analysis reveals that CH_3_OH production, requiring a six-electron transfer process, exhibits lower selectivity in photocatalytic CO_2_ reduction compared to two-electron products like CO and HCOOH [7].

In recent years, transition metal disulfide (TMD) catalysts such as MoS_2_ and WS_2_ have received widespread attention due to their high performance and low cost [8,9,10,11]. One study demonstrates that the strong interfacial coupling between exsoluted CdS and its support matrix ensures exceptional cycling stability under both UV–visible and visible light irradiation. Notably, the system achieves an outstanding hydrogen evolution rate of 3050 μmol g^−1^ h^−1^ under visible light, showcasing its superior photocatalytic performance [12]. Furthermore, Ding et al. demonstrated that 1T-WS2 significantly enhances ZnIn2S4’s photocatalytic performance by increasing catalytic active sites and photogenerated charge separation efficiency. The modified system exhibits optimised reduction potential with more favourable H* intermediate adsorption thermodynamics, as evidenced by reduced Gibbs free energy. These synergistic enhancements result in substantially improved hydrogen evolution rates under both simulated solar irradiation and 370 nm monochromatic light illumination [13]. Researchers modulate surface defects in TMDs to synergistically enhance light absorption, carrier separation efficiency, and CO_2_ reactivity [14,15,16]. One study shows that in the photoreduction of CO_2_ to CH_4_, the SVs in the catalyst Cu_3_SnS_4_ lead to the appearance of Cu(I) and Sn(II), which successfully inhibits electron–hole recombination and improves reaction activity and selectivity [17]. Among various TMDs, MoS_2_ has been widely employed for constructing SV-modified CO_2_ photoreduction catalysts due to its cost-effectiveness, facile synthesis, and tunable electronic structure [18,19]. Therefore, the introduction of SVs effectively modulates both the band structure and surface electronic states of MoS_2_, reducing the adsorption free energy of CO_2_ and intermediates while establishing an electronic foundation for selective CH_3_OH production through deep CO_2_ reduction [20]. Zhang et al. revealed that Cu doping in MoS_2_ selectively modifies different SV types: edge vacancies (active for CH_4_ formation) and basal plane vacancies (active for CH_3_OH production). Their work demonstrated that preferential passivation of edge vacancies by Cu doping significantly enhances methanol selectivity while suppressing methane generation [21].

Furthermore, the construction of heterojunctions is also widely recognised as a method to improve catalyst performance. It has been shown that heterojunctions can effectively form charge-separated interfaces that provide the driving force for electron-selective transfer to extend the photogenerated carrier lifetime [22,23,24]. In TMDs, MoS_2_ shows great potential in constructing efficient S-scheme heterojunction photocatalysts with g-C_3_N_4_ due to its narrow band gap and wide range of photoresponsivity [25,26]. Bashal et al. developed a g-C_3_N_4_/MoS_2_/Cu heterostructured catalyst, where the S-scheme structure of g-C_3_N_4_/MoS_2_ improves CO_2_ adsorption and diffusion properties and provides additional surface active sites for higher redox capacity [27]. Research has demonstrated that the heterojunction can efficiently capture photoexcited electrons from MoS_2_. Additionally, the localised electric field generated at the heterojunction interface facilitates the transfer of photogenerated electrons from MoS_2_ to g-C_3_N_4_, thereby extending their relaxation time [28]. Furthermore, Cui et al. developed a MoS_2_-45@C catalyst designed to expose additional active sites. In this system, MoS_2_ formed strong interactions with the sulphur- and nitrogen-doped carbon capping layer, enhancing CO_2_ adsorption capacity and reducing the Gibbs free energy of hydrogen adsorption. These modifications facilitated CO_2_ hydrogenation, ultimately improving methanol selectivity [29].

Although defect engineering in TMDs has been proposed as a strategy to enhance CO_2_ reactivity, and their heterostructures have demonstrated improved photogenerated charge separation, the underlying mechanism by which defect modulation in TMDs synergistically interacts with S-scheme heterojunctions to optimise CH_3_OH selectivity remains insufficiently explored. Therefore, further exploration of the photocatalytic potential of TMDs and the development of synergistic photocatalytic systems featuring efficient charge separation, enhanced CO_2_ activation, and multi-electron transfer capabilities is of critical importance.

In this study, we propose a dual-mechanism design strategy involving synergistic defect modulation and interfacial band engineering. This approach combines the introduction of SVs in MoS_2_ to enhance CO_2_ adsorption and electron enrichment with the construction of an S-scheme heterojunction with g-C_3_N_4_, achieving coordinated optimisation of photocatalytic performance. This structure promotes interfacial directed charge migration, lowers the reaction barrier, and enhances the selectivity of the deeply reduced product (CH_3_OH) while maintaining strong redox capacity. Through comprehensive structural characterisation, photoelectron spectroscopy, in situ spectroscopic analysis, and energy band modelling, this study elucidates the fundamental mechanism by which vacancy-heterojunction synergy directs the CO_2_ reduction pathway. This work establishes both a theoretical framework and a practical blueprint for developing efficient multielectron CO_2_ reduction catalysts, while simultaneously advancing innovative design principles for photocatalytic interfacial engineering.

## 2. Materials and Methods

### 2.1. Synthesis of Catalysts

In this study, g-C_3_N_4_ was synthesised through thermal polycondensation of urea. Specifically, 10 g of urea was placed in a covered alumina crucible and heated in a muffle furnace under ambient atmosphere. The temperature was raised to 550 °C at a heating rate of 5 °C min^−^**^1^** and maintained for 3 h. After natural cooling to room temperature, a light-yellow g-C_3_N_4_ powder was obtained [30].

MoS_2_ was synthesised by a hydrothermal method: A total of 0.5 mmol of (NH_4_)_6_Mo_7_O_24_-4H_2_O was dissolved with 2.5 mmol of thiourea in 40 mL of deionised water. After magnetic stirring for 30 min, the sample was transferred to a 100 mL PTFE-lined reactor and reacted at 200 °C for 24 h. The resulting precipitate was washed by centrifugation and dried as a MoS_2_ sample. In order to construct the SV structure, the resulting MoS_2_ was heat-treated at 500 °C for 2 h under Ar atmosphere to produce MoS_2_-SVs [31].

The synthesis of MoS_2_/g-C_3_N_4_ and MoS_2_-SVs/g-C_3_N_4_ S-scheme heterojunctions was carried out by an ultrasound-assisted mixing method. The two components with a certain mass ratio (MoS_2_–g-C_3_N_4_ = 1:3) were dispersed in ethanol, ultrasonically dispersed for 30 min, and then magnetically stirred for 12 h. The resultant mixture was dried at 60 °C and then annealed at 300 °C for 2 h (N_2_ atmosphere), to obtain the final composite, MoS_2_/g-C_3_N _4_ with MoS_2_-SVs/g-C_3_N_4_ (abbreviated as MoS_2_/CN and MoS_2_-SVs/CN).

### 2.2. Structural Characterisation of Catalysts

The samples’ morphological and microstructural features were examined using transmission electron microscopy (TEM, JEOL JEM-2100F, JEOL Ltd., Tokyo, Japan) and high-resolution TEM (HRTEM). Elemental distributions were acquired through high-angle annular dark-field scanning TEM (HAADF-STEM) coupled with energy-dispersive X-ray spectroscopy (EDS) mapping. Crystalline phases were determined by X-ray diffraction (XRD, Bruker D8 Advance, Bruker AXS GmbH, Karlsruhe, Germany) with a range of 5–80° [32].

The surface chemical state of the materials was analysed by X-ray photoelectron spectroscopy (XPS, Thermo Scientific ESCALAB 250Xi, Thermo Fisher Scientific Inc., Waltham, MA, USA), combining high-resolution Mo 3d, S 2p, and N 1s spectra for quantitative assessment of vacancy introduction and interfacial electronic structure changes. Fourier transform infrared spectroscopy (FT-IR) was used to detect functional group composition. Ultraviolet–visible diffuse reflectance spectroscopy (UV-Vis DRS, UV-2600 spectrophotometer, Shimadzu Corp., Kyoto, Japan) was used to determine the absorbance range of the samples and to estimate the band gap width. And photoluminescence spectroscopy (PL, Horiba Fluoromax-4, Horiba Scientific, Kyoto, Japan) was used to evaluate the photogenerated carrier complex behaviour.

### 2.3. Photocatalytic Performance Test

The CO_2_ photoreduction performance was evaluated in a closed-cycle gas–solid reaction system. The reactor consisted of a quartz photocatalytic chamber with a top quartz window for light introduction. For the experiment, approximately 20 mg of catalyst was evenly spread in a quartz dish at the reactor bottom. Before irradiation, the system was continuously purged with a CO_2_/H_2_O gas mixture (4:1 volume ratio) for 30 min to establish the initial reaction atmosphere. During the reaction, a 300 W xenon lamp (PLS-SXE300, Perfectlight) served as the light source, providing an intensity of 100 mW·cm^−2^ (measured by a CEL-NP2000 power meter) with a wavelength range of 320–780 nm to simulate sunlight conditions.

The reaction duration of a single experiment was 4 h, and the products were subsequently quantified by gas chromatography (GC-2030, Shimadzu). The detectors used included a hydrogen flame ionisation detector (FID) and a thermal conductivity detector (TCD) for the detection of the reduction products such as CH_4_, CH_3_OH, and CO. Each set of experiments was repeated three times and the average value was taken as the final result.

## 3. Results and Discussion

### 3.1. Structural and Optical Characteristics

**Figure 1:**
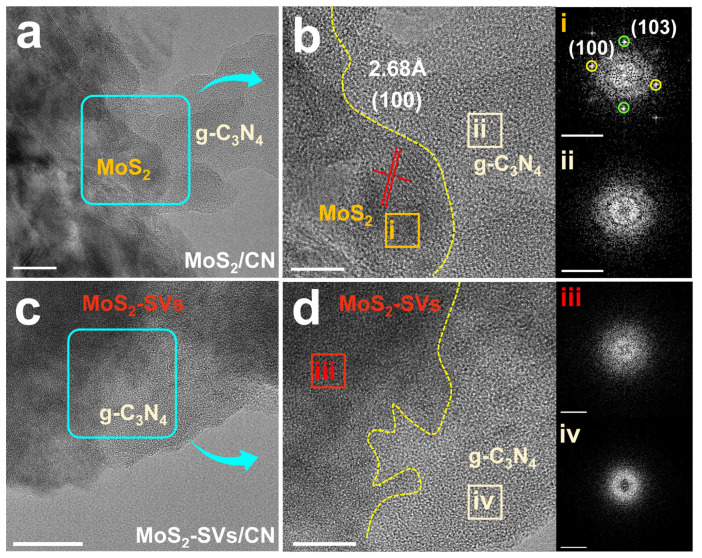
(**a**) TEM and (**b**) high-resolution TEM images of MoS_2_/CN with FFT patterns. (**c**) TEM and (**d**) high-resolution TEM images of MoS_2_-SVs/CN with FFT patterns.

Figure 1 illustrates HRTEM images of MoS_2_/CN and MoS_2_-SVs/CN samples. In Figure 1a, it can be observed that MoS_2_ in MoS_2_/CN is tightly bound to g-C_3_N_4_, constituting a typical lamellar heterostructure. The MoS_2_ nanosheets are uniformly dispersed on the g-C_3_N_4_ substrate, maintaining a well-defined flake-like morphology with flat interfacial contact. No significant agglomeration or delamination is observed, demonstrating effective microscopic-scale interfacial coupling between the two materials. This face-to-face contact geometry facilitates efficient interfacial electron transfer, establishing an ideal structural foundation for developing the efficient charge separation characteristics of the S-scheme heterojunction.

The structure is further enlarged as shown in Figure 1b, where the MoS_2_ region exhibits clear lattice striations with a measured crystal spacing of 2.68 Å, corresponding to its (100) crystal surface [33,34]. These observations indicate that the synthesised MoS_2_ possesses excellent crystallinity, characterised by well-ordered lattice fringes and smooth phase boundaries, while maintaining intimate interfacial contact with the adjacent g-C_3_N_4_ matrix. The inset exhibits multiple well-defined spots, further confirming the crystal ordering of the MoS_2_ region. It is worth pointing out that the g-C_3_N_4_ region, in contrast, does not exhibit significant crystalline features, which is in line with its intrinsic low-crystallinity structural properties.

In Figure 1c, the low-magnification HRTEM image of MoS_2_-SVs/CN demonstrates a lamellar heterogeneous structure similar to that of the control sample. However, the edges of the MoS_2_-SVs region are clearly blurred, showing a mildly diffuse profile. This structural difference can be attributed to the introduction of SVs leading to the formation of localised disorder or amorphous cladding. The amorphous nature typically introduces a high density of unsaturated coordination sites and electronic state modifications, which collectively enhance CO_2_ adsorption and activation while providing additional active sites for subsequent catalytic processes.

Figure 1d shows the high-resolution structural map of MoS_2_-SVs/CN, which further reveals the significant changes in the crystal structure of MoS_2_ surface. It is observed that the MoS_2_-SVs region no longer exhibits clear lattice stripes but exhibits a typical disordered amorphous structure. The boundary regions also show obvious blurring, confirming the surface reconstruction phenomenon induced by SVs. The FFT spectra in the inset lack clear diffraction spots and exhibit only a slight diffuse background signal, further confirming the presence of an amorphous shell layer. Meanwhile, the g-C_3_N_4_ region remains in its original low-crystalline state, unaffected by the vacancy treatment. The construction of this amorphous–crystalline interface is expected to form an electron-rich region and charge-separation-driven sites at the interface, which provides key support for efficient and selective CH_3_OH generation.

**Figure 2:**
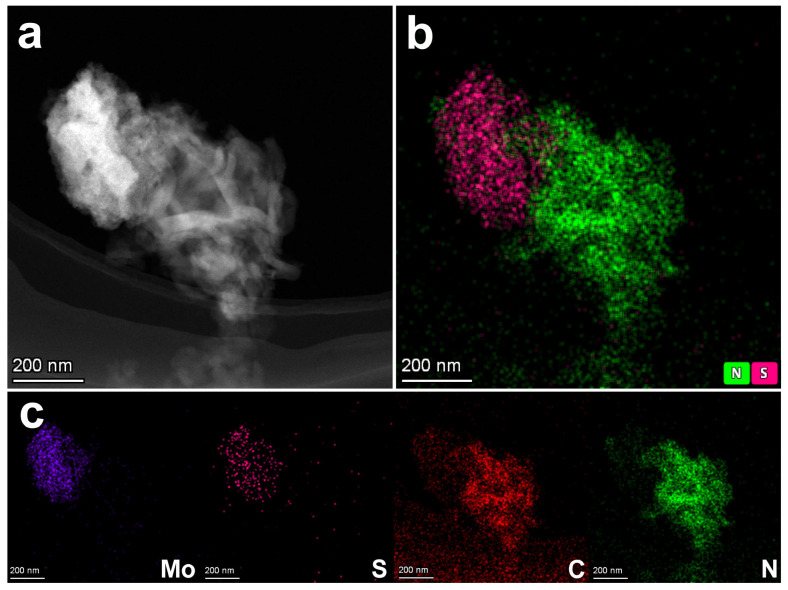
(**a**) HAADF-STEM image of MoS_2_-SVs/CN. (**b**) Overlay elemental mapping of N (green) and S (pink). (**c**) Elemental maps of Mo, S, C, and N.

Figure 2a presents the HAADF-STEM image of MoS_2_-SVs/CN, revealing a cluster-like stacked morphology. The material primarily consists of interwoven lamellar sheets, exhibiting relatively indistinct boundary features. The difference in brightness arises from the difference in the atomic number of the different elements, where elements with higher atomic numbers (Mo) show higher brightness in the diagram. This morphological feature suggests that the samples form well-organised nanoscale assemblies and indicates the presence of potential interfacial regions that facilitate directional electron migration and interfacial reactions. The overlay elemental mapping of nitrogen (N) and sulphur (S) is displayed in Figure 2b. The green signal corresponds to nitrogen (N), predominantly distributed in the sample’s right region, marking the spatial domain of g-C_3_N_4_. In contrast, the pink signal represents sulphur (S), which is primarily concentrated on the left side, delineating the MoS_2_ distribution area. The existence of crossover region between the two at the interface suggests that MoS_2_-SVs and g-C_3_N_4_ are effectively complexed at the nanoscale, forming an actual contact interface. This heterogeneous binding mode is of key importance for photogenerated carrier separation and interfacial transfer.

The further elemental distribution is shown in Figure 2c, where all four elements, Mo, S, C, and N, are effectively separated and labelled. Mo (purple) is concentrated in the left region, showing spatial consistency with S, confirming this area as the MoS_2_ phase. C (red) and N (green) are distributed in the right region, corresponding to the g-C_3_N_4_ framework. The clear boundaries and moderate signal overlap between different elements reflect that MoS_2_-SVs and g-C_3_N_4_ achieve a composite configuration with clear components and inter-embedded interfaces, which can help to enhance the structural stability and reaction synergy of the catalysts.

### 3.2. Morphological and Structural Characterisation

**Figure 3:**
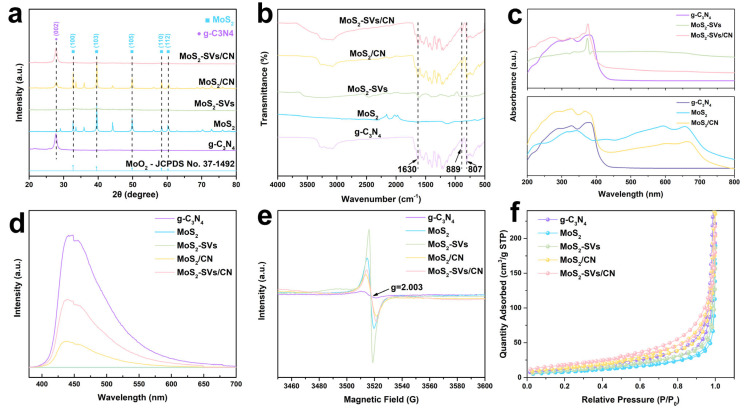
(**a**) XRD patterns, (**b**) FT-IR spectra, (**c**) UV–vis absorption spectra, (**d**) photoluminescence, (**e**) EPR, and (**f**) BET spectra of g-C_3_N_4_, MoS_2_, MoS_2_-SVs, MoS_2_/CN, and MoS_2_-SVs/CN.

Figure 3a presents the XRD patterns of the samples. The g-C_3_N_4_ displays a strong (002) diffraction peak at 27.4°, characteristic of its layered aromatic heterocyclic stacking structure. Meanwhile, MoS_2_ shows distinct peaks at 14.4° (002), 33.1° (100), and 39.6° (103), confirming the hexagonal 2H-MoS_2_ phase [35,36]. The MoS_2_-SVs samples maintain diffraction peaks identical to pristine MoS_2_, with no impurity phases detected, confirming that SV introduction preserves the host crystal structure. Notably, the MoS_2_-SVs/CN composite exhibits characteristic peaks from both g-C_3_N_4_ and MoS_2_ components without additional diffraction signals, demonstrating their physical combination as a heterostructure without new phase formation.

The Fourier transform infrared (FT-IR) spectra of the five groups of samples are shown in Figure 3b. g-C_3_N_4_ exhibits typical absorption bands in the range of 800–1800 cm^−1^, of which 1630 cm^−1^ is the peak of the C=N telescoping vibration, and 889 cm^−1^ and 807 cm^−1^ are respiratory vibrational peaks attributed to the triazine ring, respectively [37,38]. The MoS_2_ and MoS_2_-SVs samples have no obvious absorption features in the mid-infrared region, showing typical inorganic sulphide features. In the MoS_2_-SVs/CN composites, however, these g-C_3_N_4_ characteristic peaks still exist and their positions are not significantly shifted, indicating that the composite process retained the original functional group structures of the components and did not form new covalent bonding connection patterns.

In Figure 3c the UV-Vis diffuse reflectance spectra (UV-Vis DRS) of the samples are compared, which can be used to assess their light absorption capacity. The absorption edge of g-C_3_N_4_ is located roughly at 460 nm, whereas MoS_2_ significantly extends its absorption range towards the visible region due to its narrow band gap property, exhibiting strong broadband absorption characteristics [39,40]. Moreover, the MoS_2_-SVs/CN composite demonstrate broadened light absorption with enhanced intensity, particularly across the 400–700 nm visible range. This spectral enhancement reveals that SV introduction improves the intrinsic photoresponse of MoS_2_ while the constructed heterojunction synergistically enhances interfacial light harvesting and energy conversion efficiency, ultimately boosting the overall photocatalytic performance.

Figure 3d further shows the photoluminescence (PL) spectra of the samples for assessing the complex behaviour of the photogenerated carriers. g-C_3_N_4_ exhibits a strong photoluminescence (PL) peak at ~455 nm, indicating a high charge recombination rate. In contrast, both MoS_2_ and MoS_2_/CN samples show significantly quenched PL intensity, demonstrating that MoS_2_ incorporation effectively suppresses electron–hole recombination [31,41]. Interestingly, the MoS_2_-SVs/CN sample has the lowest PL emission intensity, indicating that the material has an optimal charge separation efficiency. This result suggests that the introduction of SVs together with the synergistic effect of the heterostructure improves the interfacial electron transfer efficiency, which is an important mechanism underlying its enhanced CO_2_ reduction performance.

The electron paramagnetic resonance (EPR) spectra reveal the presence of unpaired electrons in all catalysts at room temperature (Figure 3e). All samples exhibit a typical signal near g = 2.003, commonly associated with surface defects or radical centres. The weak EPR signals of pure MoS_2_ and g-C_3_N_4_ suggest a low intrinsic defect concentration. In contrast, the MoS_2_-SVs sample displays a markedly enhanced EPR intensity, directly indicating the successful introduction of sulphur vacancies. The MoS_2_/CN composite shows slightly stronger signals than the individual components, implying that heterojunction formation induces limited electronic redistribution and defect generation. Notably, MoS_2_-SVs/CN exhibits the strongest EPR signal, reflecting the highest concentration of sulphur vacancies and the most significant electronic structure perturbation. These vacancies serve as effective sites for charge carrier trapping and separation, thereby playing a crucial role in enhancing photocatalytic performance.

Nitrogen adsorption–desorption isotherms of g-C_3_N_4_, MoS_2_, MoS_2_-SVs, MoS_2_/CN, and MoS_2_-SVs/CN catalysts are shown in Figure 3f. All samples exhibit typical Type IV isotherms with pronounced hysteresis loops in the high relative pressure range, indicative of well-developed mesoporous structures. Compared with the single-component materials, the formation of heterojunctions significantly enhances the pore structure. Specifically, MoS_2_/CN shows a markedly higher adsorption capacity than pure g-C_3_N_4_ and MoS_2_, suggesting that the introduction of MoS_2_ inhibits layer stacking and promotes interlayer exposure. Upon further incorporation of sulphur vacancies, the MoS_2_-SVs/CN sample exhibits the highest nitrogen uptake, indicating that defect engineering effectively tunes the surface and pore architecture of the catalyst. This structural optimisation not only increases the specific surface area and porosity but also facilitates the rapid mass transfer of CO_2_ and H_2_O molecules, thereby providing more accessible pathways to active sites and promoting photocatalytic efficiency.

### 3.3. Photocatalytic Performance Evaluation

**Figure 4:**
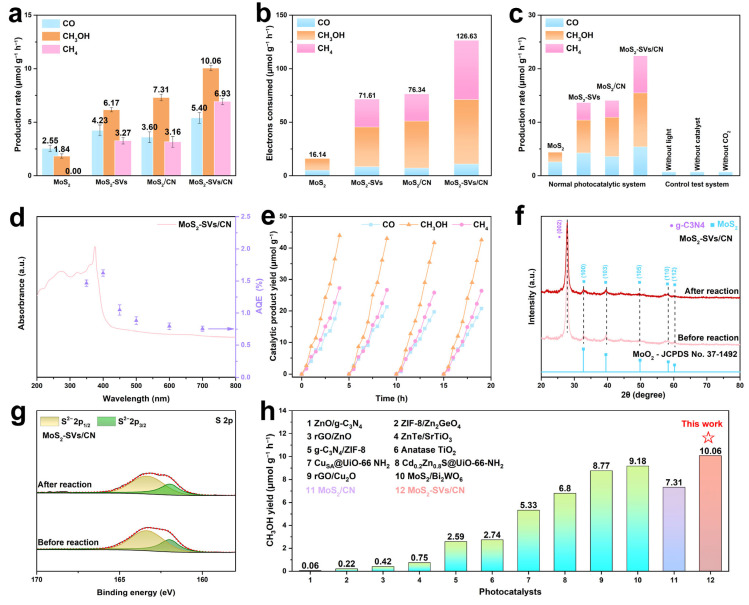
(**a**) Photocatalytic CO_2_ reduction rates and (**b**) electron consumption amounts of different samples. (**c**) Comparison of product rates under standard and control conditions. (**d**) AQE of MoS2-SVs/CN at different irradiations. (**e**) Time-dependent product yields of MoS_2_-SVs/CN. (**f**) XRD patterns and (**g**) S 2p spectra of MoS_2_-SVs/CN before and after photocatalysis. (**h**) Comparison of CH_3_OH production rates with representative photocatalysts.

In Figure 4a, both MoS_2_ and its modified samples produce three types of reduction products, CO, CH_4_, and CH_3_OH, in the CO_2_ photoreduction reaction, which were systematically detected and compared after a single experiment Among all catalysts, MoS_2_ produced almost no CH_4_ during CO_2_ photoreduction and the CO and CH_3_OH yields were very low, only 2.55 and 1.84 μmol·h^−1^·g^−1^, which can be attributed to its limited CO_2_ activation capability and rapid charge recombination. In comparison, the MoS_2_-SVs treated by the defective project significantly enhanced the reduction capacity with the generation of all three products; especially, the CH_3_OH yield was 3.35 times higher than that of the untreated MoS_2_, due to the creation of defect states that lowered the conduction band position and facilitated multi-electron transfer processes. When MoS_2_ and g-C_3_N_4_ constructed a heterostructure (MoS_2_/CN), the CH_3_OH generation was further elevated to about 7.31 μmol·h^−1^·g^−1^, whereas the yields of the remaining two products were slightly decreased as a result of efficient interfacial charge separation driven by the built-in electric field at the heterojunction. MoS_2_-SVs/CN achieved the maximum yield of all three products, with CH_3_OH as high as 10.06 μmol·h^−1^·g^−1^, where the SVs served as active sites for stabilising key intermediates (*COOH) while the S-scheme charge transfer mechanism maintained strong reduction potential for selective CH_3_OH formation through a proton-coupled electron transfer pathway.

Figure 4b analyses the catalytic performance of each catalyst in terms of electron consumption. MoS_2_ had the lowest electron utilisation efficiency of 16.14 μmol·h^−1^·g^−1^, suggesting that most of the generated photogenerated electrons may have undergone ineffective complexation. Comparatively, both MoS_2_-SVs and MoS_2_/CN samples showed significant improvement in electron transfer efficiency. In particular, MoS_2_-SVs/CN, with a total electron consumption of up to 126.63 μmol·h^−1^·g^−1^, showed the best performance among all samples. It is worth emphasising that the vast majority of these electrons are used for the generation of CH_3_OH, highlighting the system’s high preference for multi-electron reduction pathways coupled with a strong electron supply capacity.

To exclude the effect of side effects, the results of multiple control experiments are given in Figure 4c. As can be seen from the figure, significant gas production behaviour of the reaction system could only be achieved when the conditions of light, CO_2_, and a catalyst are present simultaneously. No products were detected under no-light, no-CO_2_, or no-catalyst conditions, which fully indicates that the reaction pathway of the present system is indeed entirely derived from photocatalytic CO_2_ reduction. Further comparison of the four catalysts, MoS_2_, MoS_2_-SVs, MoS_2_/CN, and MoS_2_-SVs/CN, reveals that the composite structure and the simultaneous introduction of the vacancies are the key to achieving high yields.

The correlation between the light absorption characteristics of the MoS_2_-SVs/CN catalyst and its apparent quantum efficiency (AQE) across different wavelengths reveals a strong dependence on excitation energy (Figure 4d). The absorption spectrum indicates that the composite catalyst exhibits strong absorption across the entire visible light region. The AQE curve shows that the catalyst reaches its maximum quantum efficiency of 1.63% under 400 nm irradiation, followed by a gradual decline with increasing wavelength. Despite the decrease, a relatively stable photocatalytic response is maintained throughout the visible range (400–700 nm), suggesting effective utilisation of visible light. This broad spectral response can be attributed to the band gap reconstruction and enhanced charge separation efficiency resulting from the introduction of SVs.

MoS_2_-SVs/CN was used to run five consecutive cycles under cyclic light/dark conditions with a reaction time of 20 h (Figure 4e). It can be observed that all three reduction products of MoS_2_-SVs/CN showed a linear increase with time in each cycle. These results demonstrate the catalyst’s exceptional stability and sustained activity throughout prolonged operation. The consistent reaction rates further rule out potential deactivation pathways such as active species leaching or structural degradation under illumination. Notably, the cumulative yield of CH_3_OH consistently surpassed that of CO and CH_4_ by a significant margin, unequivocally confirming the system’s high selectivity toward deeply reduced products and its suitability for long-term, high-load CO_2_ conversion applications.

To evaluate the structural stability of the catalyst, XRD patterns of the MoS_2_-SVs/CN composite before and after the photocatalytic reaction were recorded (Figure 4f). The diffraction peaks remain nearly unchanged after the reaction, suggesting robust structural retention. A distinct peak at 27.4° corresponds to the (002) plane of g-C_3_N_4_, serving as a fingerprint of its layered framework. Meanwhile, multiple reflections from MoS_2_ are still present, confirming the absence of any phase transformation throughout the catalytic process. Figure 4g presents the high-resolution XPS spectra of S 2p before and after the reaction. In both cases, two typical S^2−^ signals are observed, with no additional peaks corresponding to high-valence sulphur species, confirming the chemical stability of the catalyst. These findings, consistent with the XRD analysis, demonstrate that the MoS_2_-SVs/CN composite exhibits excellent structural and chemical stability during the photocatalytic CO_2_ reduction process. Moreover, the preserved SVs suggest their sustained involvement in electronic modulation throughout the reaction. Furthermore, Figure 4h presents a comparative analysis of CH_3_OH production rates between the MoS_2_-SVs/CN catalyst and other representative systems, clearly demonstrating its superior performance [42,43,44,45,46,47,48,49,50,51]. The catalyst achieves exceptional yields through synergistic TMD modification and heterojunction engineering, underscoring the significant advancement of this design for photocatalytic CO_2_-to-CH_3_OH conversion.

### 3.4. Electronic Structure and Band Modulation

**Figure 5:**
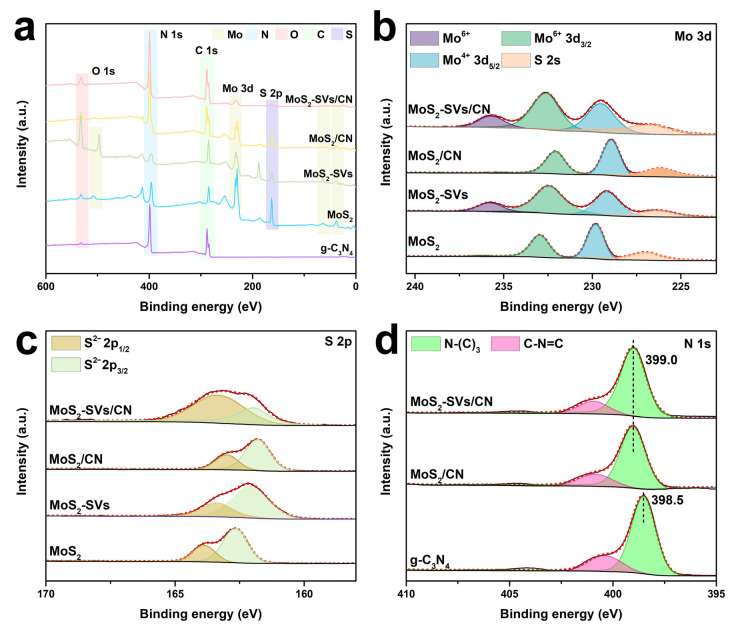
(**a**) XPS survey spectra of g-C_3_N_4_, MoS_2_, MoS_2_-SVs, MoS_2_/CN, and MoS_2_-SVs/CN. (**b**) High-resolution Mo 3d spectra. (**c**) S 2p spectra confirming the presence of SVs. (**d**) N 1s spectra of g-C_3_N_4_-based samples.

Figure 5a shows the full XPS spectra of each sample, giving a comprehensive picture of the presence of the elements Mo, S, C, N, and O. The g-C_3_N_4_ samples exhibit significant C 1s and N 1s signals, while strong Mo 3d and S 2p peak positions are observed in the MoS_2_ samples, the combination of which constitutes the basic elemental framework of the heterogeneous structure. With the gradual complexity of the structural combinations, four types of characteristic signals of Mo, S, C, and N appear simultaneously in the MoS_2_-SVs, MoS_2_/CN, and MoS_2_-SVs/CN samples, which indicates that the components in the heterostructures were successfully retained and complexed with each other [35,52]. In addition, a weak O 1s peak appears in MoS_2_-SVs/CN, which is speculated to be possibly derived from surface hydroxyl groups or adsorbed oxygen, which are oxygen functional groups that may provide additional surface active sites during CO_2_ reduction.

The high-resolution spectrum of the Mo 3d orbital is illustrated in Figure 5b. Two pairs of typical double peaks, Mo^4+^ 3d_5/2_ (~229.5 eV) and Mo^6+^ 3d_3/2_ (~232.7 eV), are observed in all the samples, representing the main valence structure of MoS_2_ [53,54]. Notably, in MoS_2_-SVs and MoS_2_-SVs/CN, the signals of the Mo^6+^ component appear to be enhanced, indicating that some of the Mo atoms were further oxidised, induced by the defects. The presence of this high-valence Mo is usually closely associated with vacancy-induced charge redistribution, contributing to the enhancement of interfacial electron affinity and CO_2_ activation.

The chemical states of elemental S are further resolved (Figure 5c). S 2p_3/2_ and S 2p_1/2_ in MoS_2_ are located at 162.3 eV versus 163.5 eV, which belongs to the typical S^2−^ state. Meanwhile, in the MoS_2_-SVs and their composite samples, the intensity of the main peak is relatively weakened and the broadening of the shoulder peak is obvious, demonstrating that part of the S^2−^ is withdrawn and structural vacancies are formed. Combined with the oxidation state change in Mo, it can be confirmed that the generation of SVs indeed triggers a reorganisation of the electronic structure, offering the possibility of modulating the reaction path and adsorption properties.

The N 1s orbitals are analysed in detail in Figure 5d to reveal the microenvironment of nitrogen in the g-C_3_N_4_ structure. The main peak in g-C_3_N_4_ is located at 398.5 eV and corresponds to the sp^2^ hybridised melamine backbone (N-(C)_3_), while the small shoulder peak corresponds to the amino site or C-N=C linkage mode [55,56]. With the introduction of MoS_2_, especially in MoS_2_-SVs/CN, the position of the N-(C)_3_ peak is positively shifted to 399.0 eV, suggesting a decrease in the electron cloud density in g-C_3_N_4_, which may be caused by interfacial electron transfer. This result is consistent with the enhanced charge separation ability derived from the PL and performance tests in Figure 3, further confirming the substantial modulation of electronic behaviour by the construction of heterostructured interfaces.

**Figure 6:**
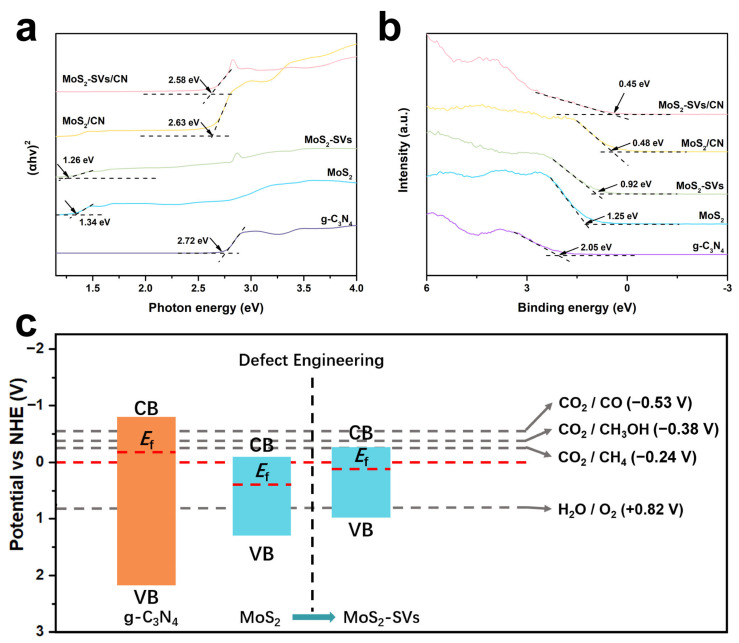
(**a**) Tauc’s plots for band gap estimation of g-C_3_N_4_, MoS_2_, MoS_2_-SVs, MoS_2_/CN, and MoS_2_-SVs/CN. (**b**) Valence bands of g-C_3_N_4_, MoS_2_, MoS_2_-SVs, MoS_2_/CN, and MoS_2_-SVs/CN. (**c**) Energy band diagram showing band alignment evolution with SV engineering.

Figure 6a estimates the band gap width for each sample by the Tauc fitting method. The band gap of g-C_3_N_4_ is about 2.72 eV, which is typical of visible light responsive broadband semiconductors, and the band gap of MoS_2_ samples is smaller, 1.34 eV, which is in line with its narrow band gap semiconductor characteristics [57,58]. In MoS_2_-SVs, the band gap is slightly narrowed to 1.26 eV, indicating that the introduction of SVs triggers the shift in the energy band edges to a certain extent, which in turn enhances its ability to absorb low-energy light. The MoS_2_/CN and MoS_2_-SVs/CN composites exhibit band gaps of 2.63 eV and 2.58 eV, respectively, representing a slight reduction compared to pristine g-C_3_N_4_. This narrowing indicates that heterojunction formation modifies the energy-level structure, generating an energetic driving force that facilitates interfacial electron transfer and promotes multi-step CO_2_ reduction.

To further resolve the variation in the Fermi energy levels with respect to the work function, the UPS spectra of the samples are presented in Figure 6b. g-C_3_N_4_ exhibits the lowest work function (~2.05 eV), facilitating electron emission and making it an effective electron donor in the heterostructure. In contrast, MoS_2_ demonstrates an elevated work function of 1.25 eV, indicating stronger electron affinity [32,59]. After introducing SVs, the work function further decreases to 0.92 eV, demonstrating a defect-induced energy level elevation effect. In the composite samples, the work functions of MoS_2_/CN and MoS_2_-SVs/CN measure 0.48 eV and 0.45 eV, respectively, significantly lower than those of individual components [35,60]. This substantial reduction provides direct electronic evidence for strengthened directional charge transfer and spatial separation at the interface, conclusively demonstrating the formation of an S-scheme heterojunction.

Figure 6c summarises the energy band structure evolution of each component and depicts the thermodynamic feasibility of its light-driven pathway in combination with the reaction potentials of CO_2_ reduction and water oxidation. In undoped defective MoS_2_, the conduction band potential is already slightly above the demand for the CO_2_/CH_4_ (−0.24 V) reaction, whereas vacancy-moderated MoS_2_-SVs exhibit higher reducing power and deeper conduction band positions. The valence band of g-C_3_N_4_ is located near +1.50 V, which is suitable for providing holes to participate in the H_2_O oxidation reaction (+0.82 V) [60,61,62]. In MoS_2_-SVs/CN heterojunctions, electrons can migrate from the g-C_3_N_4_ conduction band to the MoS_2_-SVs conduction band, while holes are retained in the g-C_3_N_4_ valence band to achieve an effective space separation. This S-scheme charge transfer mechanism not only ensures efficient photogenerated carrier utilisation but also provides sufficient electron accumulation and supply conditions for multi-electron reaction pathways (CO_2_ to CH_3_OH).

**Figure 7:**
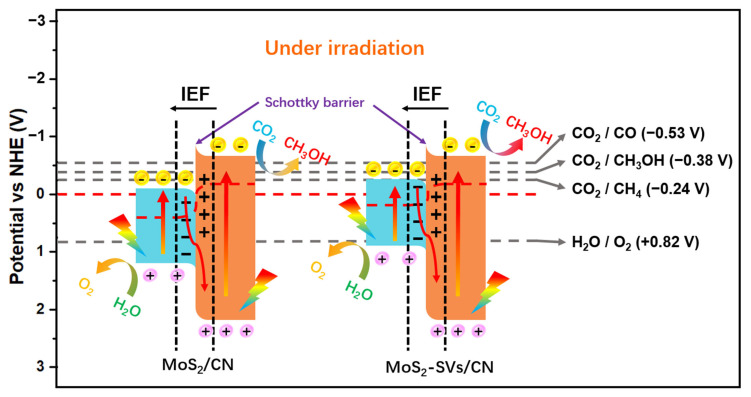
Schematic charge transfer mechanism under light irradiation in MoS_2_/CN and MoS_2_-SVs/CN systems.

Figure 7 presents a mechanistic schematic illustrating the charge behaviour in MoS_2_/CN and MoS_2_-SVs/CN photocatalytic systems under illumination. The diagram visually elucidates how heterointerface engineering directs photogenerated carrier migration pathways and governs product selectivity. In both structures, an S-scheme energy band arrangement is formed between g-C_3_N_4_ and MoS_2_, allowing electrons to be injected from the g-C_3_N_4_ conduction band to the MoS_2_ side, while the holes are retained in the valence band of g-C_3_N_4_. Due to the significant energy level difference between the two phases, the interfacial built-in electric field (IEF) is established, which drives the efficient spatial separation and directional migration of photogenerated carriers.

In the MoS_2_/CN system, the intrinsically low defect density of MoS_2_ limits electron concentration accumulation despite its thermodynamically favourable conduction band position for reduction. Consequently, electron injection capacity remains constrained even after Schottky barrier formation. This to some extent constrains the chances of deep reduction pathways (CH_3_OH or CH_4_) occurring, with the product distribution being more favourable for the single-electron reduction product CO. In contrast, the MoS_2_-SVs/CN system exhibits significant electronic structure modulation through SVs engineering. This modification induces a pronounced negative shift in the conduction band position and enhances charge carrier enrichment, thereby enabling more electrons to overcome potential barriers and participate in reduction reactions.

It is worth mentioning that in MoS_2_-SVs/CN, the enhanced interfacial built-in electric field (IEF) together with the lower barrier height promotes the fast migration and stable enrichment of electrons at the interface, thus providing a continuous electron flow for the multi-electron reduction of CO_2_. The corresponding product distribution, in which CH_3_OH becomes the major product, is a direct reflection of the enhanced reaction path selectivity brought about by this interfacial energy band modulation strategy. In addition, the oxidation reaction occurs mainly on the MoS_2_-SVs side, whose valence band potential is higher than the H_2_O/O_2_ oxidation potential (+0.82 V), which can provide sufficient driving force for photogenerated holes to achieve O_2_ generation [63].

## 4. Conclusions

In this study, MoS_2_-SVs/g-C_3_N_4_ S-scheme heterojunction photocatalysts were constructed by introducing SVs to achieve a synergistic enhancement of the selectivity and efficiency of the CO_2_ photoreduction reaction. The introduction of SVs effectively modulates the electronic structure and surface coordination environment of MoS_2_, which not only increases the density of unsaturated active sites but also induces a downward shift in its conduction band and enhances the adsorption and activation of CO_2_. Upon complexation with g-C_3_N_4_ to form an S-scheme heterojunction, the interfacial electron redistribution significantly contributes to the spatial segregation of photogenerated carriers in the heterostructure. Structural and surface analyses of MoS_2_-SVs/g-C_3_N_4_ confirmed that the heterostructure has close interfacial contacts, homogeneously dispersed components, and stable vacancy states. Optoelectronic analyses revealed the establishment of an S-scheme band alignment with a built-in electric field, which effectively suppresses charge recombination while enabling directional carrier migration. The optimised catalyst achieved an exceptional CH_3_OH production rate of 10.06 μmol·h^−1^·g^−1^ under simulated solar irradiation. Energy band structure analysis Optoelectronic analyses revealed the establishment of an S-scheme band alignment with a built-in electric field, which effectively suppresses charge recombination while enabling directional carrier migration. This work proposes a synergistic strategy integrating defect regulation and band alignment to boost charge transport and CO_2_ selectivity, offering a practical design framework for efficient CO_2_ photoreduction involving complex electron transfer steps.

## Data Availability

The data that support the findings of this study are available from the corresponding author upon reasonable request.
